# Editorial: Advances in the Pathogenesis of Canine Neurologic Disease

**DOI:** 10.3389/fvets.2020.00623

**Published:** 2020-09-04

**Authors:** Jey W. Koehler, Andrew D. Miller

**Affiliations:** ^1^Department of Pathobiology, Auburn University College of Veterinary Medicine, Auburn, AL, United States; ^2^Department of Biomedical Sciences, Section of Anatomic Pathology, Cornell University College of Veterinary Medicine, Ithaca, NY, United States

**Keywords:** canine, neuropathology, glioma, MRI, immunohistochemistry

Veterinary neuropathology, in both the clinical and experimental realm, is a specialized field that has seen expansive growth over the last decade. This growth has led to significant advances in many aspect of clinical neurology and neuropathology and includes related areas of study including neurobehavior, neurosurgery, neuroimmunology, neuroradiology, and others. Emblematic of these research advances include updated diagnostic criteria and molecular classification of canine glioma, increased insight into infectious and non-infectious causes of meningoencephalitis in the dog, and the unraveling of a number of neurodegenerative conditions, including inherited central and peripheral nervous system diseases, lysosomal storage diseases, and age-associated neurocognitive decline in the dog. The papers presented in this collection offer additional details on several important diseases in the dog and aim to provide further details about the pathogenesis and diagnostic features of these canine nervous system diseases.

An important contribution to the comparative neuropathology literature comes from Story et al. in which the authors provide a detailed and comprehensive review of various inherited musculoskeletal and neurodegenerative diseases. This paper illuminates several important aspects of comparative canine neuropathology, especially in the emerging disease entity of canine cognitive dysfunction (CCD). Canine cognitive dysfunction is an increasingly studied disease that has some correlates to cognitive-related decline in humans. There is exceptional interest in better understanding this disease in the dog, how to ameliorate the clinical signs associated with CCD, and how it can be used to provide clues to human age-associated cognitive decline. In addition, this article provides an in depth comparative review of several common, but currently incurable degenerative conditions of the canine CNS, namely canine degenerative myelopathy, Duchenne's muscular dystrophy, and a number of lysosomal storage diseases like mucopolysaccharidosis, fucosidosis, and neuronal ceroid lipofuscinosis. These diseases are highlighted by past, present, and future therapeutic goals.

Intracranial neoplasia is a rapidly emerging area of interest in comparative pathology, especially in comparing brain tumors in dogs and people in order to better understand the ways in which we can (and cannot) use canine patients as models for the human disease. Three of the articles in this focused issue are directed at answering some of the comparative neurooncology questions that the field has posed and serve as timely follow-up articles to the recent literature on intracranial neoplasia in the dog. An article by Koehler et al. explores the influence of hypoxia on miRNA-mediated gene regulation in canine glioma cell lines. This article provides evidence that hypoxia is an important factor in the pathogenesis of canine glioma, similar to human glioma. This highlights a critical comparative aspect between canine and human glioma. In human glioma, hypoxia is linked to a poor response to treatment in some cases and if a similar feature is found consistently in canine glioma, it may provide clues to treatment outcomes and prognosis. An article by Demeter et al. provides data of widespread immunohistochemical expression of the neuronal marker MAP2 in canine glioma. This has long been described in human glioma and has been used as evidence for these tumors being derived from pluripotential progenitor cells. MAP2 has variable expression patterns in human astrocytoma and oligodendroglioma. In this article, a variation in immunolabeling was found when comparing canine astrocytoma to oligodendroglioma, similar to that described in human glioma. As brain tumor biopsy becomes more commonplace in the dog, studies like this are important as they provide avenues for future prospective studies that can establish a more refined diagnosis for dogs afflicted with glioma. And finally an article by Dalton et al. provides the first characterization of the immune cell microenvironment in canine choroid plexus neoplasms. In these tumors, B and T cells are common and large numbers of macrophages are noted in the tumor, although absent or low numbers of intravascular Mac387^+^ monocytes were noted. The immune cell population is an important facet of cancer pathobiology and has been neglected in many veterinary neoplasms. The importance of the immune cell microenvironment lies in the fact that it has been show to correlate with treatment outcome in a number of human neoplasms. While prognostic studies comparing the cancer immune cell microenvironment are generally lacking in the dog, exploratory studies like the one highlighted here provide important descriptive information for future studies. The immune cell microenvironment has previously been studied in canine meningioma and oligodendroglioma; however, studies remain scant in this area with much more work needing to be done.

Last in this collection of articles is a case series of canine snake-eye myelopathy (Rossmeisl et al.) which provides the first comprehensive clinical, imaging, and pathologic assessment of this enigmatic condition in the dog. This condition presents with bilaterally symmetric regions of T2 hyperintensities on MRI, most noticeable in the ventral horn gray matter. The geographical distribution of this lesion suggest a vascular etiology; however, the entire etiopathogenesis remains to be determined.

Canine neuropathology is a field that continues to grow with many unanswered questions. While lysosomal storage diseases, degenerative myelopathy, and intracranial neoplasms often receive the lion's share of research and publications, there are many inherited and spontaneous conditions of the canine central nervous system that deserve in depth experimental studies. These future studies will not only continue to guide the field of canine neuropathology forward, it will also open exciting new research paths that will undoubtedly synergize with and stimulate the field of comparative neuropathology.

## Author Contributions

All authors listed have made a substantial, direct and intellectual contribution to the work, and approved it for publication.

## Conflict of Interest

The authors declare that the research was conducted in the absence of any commercial or financial relationships that could be construed as a potential conflict of interest.

